# Health Literacy as a Modifiable Clinical Risk Factor: Operationalizing an Underrecognized Determinant of Outcomes

**DOI:** 10.7759/cureus.105639

**Published:** 2026-03-22

**Authors:** Cosmas C Ofoegbu

**Affiliations:** 1 Health Sciences, Central Washington College of Nursing Science, Enugu, Enugu, NGA; 2 Family Medicine, Fort St. John Hospital, Fort St. John, CAN

**Keywords:** chronic disease management, clinical risk factor, health equity, health literacy, patient adherence

## Abstract

Health literacy has been considered a social determinant of health and has never been extensively incorporated in the general practice of clinical care. Systematic reviews and cohort studies show consistent evidence of low health literacy as a correlate of worse outcomes of chronic disease, enhanced use of health care, and greater mortality. Even with this evidence, health literacy is not often screened for, documented, or even included in clinical risk stratification. This article contends that health literacy ought to be re-conceptualized as a clinical risk factor, which can be modified just like other routinely managed issues, like smoking or blood pressure. Universal communication precautions and teach-back, as literacy-sensitive strategies implementable in clinical workflow, would enhance adherence, reduce complications, and allow achieving more equitable care. The acknowledgment of health literacy as a quantifiable and alterable risk factor can be used to endorse the educational interventions as a core element of efficient chronic disease management.

## Editorial

Modern medicine has become highly effective at detecting and modifying clinical risk factors. Smoking, obesity, hypertension, alcohol consumption, and dyslipidemia are routinely screened, documented, and addressed in clinical care. These factors are embedded in clinical guidelines, quality metrics, and reimbursement frameworks. However, despite being widely recognized as a social determinant of health, health literacy is rarely operationalized within routine clinical practice and is seldom incorporated into formal clinical risk stratification models.

Health literacy broadly refers to the capacity of individuals to obtain, process, and understand health information needed to make appropriate health decisions. Sørensen and colleagues conceptualized health literacy as a multidimensional construct that includes the abilities to access, understand, appraise, and apply health information within healthcare, disease prevention, and health promotion contexts [1]. Although the concept is well established in public health literature, its integration into everyday clinical workflows remains limited.

Low health literacy is common and has substantial clinical implications. Evidence consistently demonstrates that patients with limited health literacy experience poorer health outcomes and greater healthcare utilization. A landmark systematic review by Berkman et al. showed that low health literacy is associated with worse disease knowledge, poorer chronic disease control, increased hospitalization, and overall poorer health status [2]. Importantly, these associations persist even after adjustment for education and socioeconomic status.

Beyond morbidity, health literacy has also been linked to mortality outcomes. In a longitudinal cohort study, Bostock and Steptoe found that low functional health literacy was independently associated with higher mortality among older adults [3]. These findings suggest that health literacy is not simply a social characteristic but rather a clinically meaningful risk factor that influences long-term outcomes.

Health literacy also plays a critical role in cardiovascular health. The American Heart Association has recognized health literacy as an important factor influencing prevention, treatment adherence, and patient engagement in cardiovascular disease management [4]. Patients must understand medication regimens, recognize symptoms, follow lifestyle recommendations, and navigate complex healthcare systems. When comprehension is limited, these processes become impaired, contributing to treatment failure and preventable complications.

Despite this evidence, health literacy is rarely addressed in clinical performance frameworks. Healthcare systems tend to prioritize measurable indicators such as blood pressure control, HbA1c levels, prescription rates, or hospital throughput. These metrics provide short-term quantifiable outcomes. In contrast, communication quality, patient understanding, and cognitive alignment between clinician and patient are less easily measured, even though they significantly influence long-term disease control. Consider the management of hypertension or diabetes. Pharmacologic therapy can be intensified through dosage adjustments or additional medications, and treatment effects can be monitored through laboratory values or vital signs. However, improved prescribing alone does not guarantee effective disease management. Patients must understand medication schedules, dosing instructions, potential side effects, and the importance of long-term adherence. Misunderstanding in any of these areas can undermine otherwise appropriate medical treatment.

Poor health literacy contributes to medication non-adherence, incorrect medication use, and failure to recognize warning symptoms. These problems often lead to emergency department visits, hospitalizations, and avoidable complications. Such outcomes represent both clinical harm and substantial healthcare costs. Addressing health literacy may therefore serve as an important strategy to improve patient outcomes while simultaneously reducing unnecessary healthcare utilization.

Encouragingly, health literacy is a modifiable factor. Interventions designed to improve communication and comprehension have demonstrated measurable benefits. One such strategy is the teach-back method, in which clinicians ask patients to repeat key instructions in their own words to confirm understanding. A systematic review by Ha Dinh and colleagues found that teach-back interventions improve patient knowledge, self-management behaviors, and adherence in individuals with chronic disease [5]. These findings suggest that relatively simple communication techniques can produce meaningful improvements in clinical care. Recognizing health literacy as a clinical risk factor would have several practical implications. First, it would encourage routine evaluation of patient understanding within clinical encounters. Just as clinicians screen for smoking or alcohol use, brief assessments of comprehension could identify individuals at risk for misunderstanding treatment plans.

Second, it would justify allocating time and resources toward patient education as a risk-modification strategy. Improved understanding of medication use, disease processes, and lifestyle recommendations may reduce medication errors, improve adherence, and decrease avoidable complications.

Third, it would facilitate measurement and accountability. Health systems increasingly rely on quality metrics and performance indicators. If literacy-sensitive interventions were documented within electronic health records, healthcare organizations could monitor their implementation and evaluate their impact on outcomes.

Importantly, approaches to health literacy should avoid stigmatizing patients. Labeling individuals as having “low literacy” may discourage engagement or create embarrassment. A more effective strategy is the universal precautions approach, which assumes that all patients may experience difficulty understanding health information. Simplified communication, plain language explanations, and confirmation of understanding should therefore be applied consistently across patient populations.

Addressing health literacy may also reduce health disparities. Vulnerable populations - including older adults, individuals with limited formal education, and marginalized communities - often experience disproportionate barriers to health comprehension. Integrating literacy-sensitive communication into clinical practice could therefore improve equity in healthcare delivery.

The broader transformation required is both conceptual and practical. Clinical effectiveness should not be measured solely by the prescription written or the laboratory value achieved, but also by whether patients understand how to implement treatment recommendations in their daily lives. The gap between medical advice and patient behavior often lies in comprehension. Historically, other risk factors underwent a similar transition from abstract concepts to operationalized clinical variables. Smoking was once considered a lifestyle choice rather than a medical risk factor. Obesity evolved from a descriptive characteristic to a coded clinical diagnosis incorporated into treatment guidelines. Health literacy may require a similar conceptual shift, from a social determinant discussed in policy frameworks to a measurable clinical parameter embedded in everyday practice.

Future research should further quantify the independent effects of health literacy on clinical outcomes and evaluate the cost-effectiveness of literacy-sensitive interventions. As evidence accumulates, guidelines and quality metrics may increasingly incorporate communication and comprehension measures.

Ultimately, outcomes in healthcare depend not only on what clinicians prescribe, but also on what patients understand. In an era of rising chronic disease burden and constrained healthcare resources, optimizing patient comprehension is not optional - it is a strategic component of effective clinical care.
